# The effect of treating hearing loss with hearing aids on plasma biomarkers of Alzheimer's disease and related dementias

**DOI:** 10.1002/dad2.70397

**Published:** 2026-06-23

**Authors:** Lachlan Cribb, Margarita Moreno‐Betancur, Julia Sarant, Rory Wolfe, Matthew Paul Pase, Gary Rance, Michelle M. Mielke, Anne M. Murray, Alice Owen, Robyn L. Woods, Zhen Zhou, Zimu Wu, Kerry M. Sheets, Trevor T.‐J. Chong, Raj C. Shah, Joanne Ryan

**Affiliations:** ^1^ School of Public Health and Preventive Medicine Monash University Melbourne Victoria Australia; ^2^ Clinical Epidemiology & Biostatistics Unit Murdoch Children's Research Institute Parkville Victoria Australia; ^3^ Biostatistics Unit, Department of Paediatrics The University of Melbourne Parkville Victoria Australia; ^4^ Department of Audiology and Speech Pathology The University of Melbourne Parkville Victoria Australia; ^5^ School of Psychological Sciences and Turner Institute for Brain and Mental Health Monash University Clayton Victoria Australia; ^6^ Department of Epidemiology and Prevention Wake Forest University School of Medicine Winston‐Salem North Carolina USA; ^7^ Berman Center for Outcomes & Clinical Research Hennepin Healthcare Research Institute Minneapolis Minnesota USA; ^8^ Department of Medicine, Geriatrics Division Hennepin Healthcare Minneapolis Minnesota USA; ^9^ Departments of Medicine and Neurology University of Minnesota Minneapolis Minnesota USA; ^10^ Department of Neurology Alfred Health Melbourne Victoria Australia; ^11^ Department of Clinical Neurosciences St. Vincent's Hospital Melbourne Victoria Australia; ^12^ Department of Family & Preventive Medicine and the Rush Alzheimer's Disease Center Rush University Medical Center, Chicago, Illinois, USA

**Keywords:** Alzheimer's disease, biomarkers, dementia, hearing aids, hearing loss, neuropathology, plasma biomarkers, target trial emulation

## Abstract

**BACKGROUND:**

Though evidence indicates that treating hearing loss with hearing aids (HAs) could reduce dementia risk, the effects on biomarkers of Alzheimer's disease and related dementias (ADRD) remain unknown.

**METHODS:**

Observational data from Aspirin in Reducing Events in the Elderly (ASPREE) study participants without dementia and with hearing problems were used. We emulated two target trials to estimate the effect of (1) new HA prescription and (2) the frequency of HA use on plasma ADRD biomarkers after 7 years using targeted maximum likelihood estimation, with multiple imputation for missing data.

**RESULTS:**

There was a median of 2842 individuals (mean 75 years, 48% female) across imputed datasets, and 735 new HA prescriptions. Estimated treatment effects were close to null for phosphorylated tau181, neurofilament light chain, glial fibrillary acidic protein, and amyloid beta 42/40. There was little evidence of effect modification (e.g., by apolipoprotein E ε4 genotype).

**DISCUSSION:**

In older people with hearing loss, HA prescription and frequency of use had minimal association with levels of ADRD biomarkers.

## Highlights

- A target trial investigating treatment of hearing loss with hearing aids was emulated.
- Minimal effects were observed on biomarkers of Alzheimer's disease neuropathology.
- Other mechanisms, like reducing social isolation, may underlie hearing aid benefits.

## 1 BACKGROUND

Treating hearing loss with hearing aids (HAs) is a promising strategy for limiting cognitive decline and preventing dementia.^1^ There are several potential mechanisms by which this benefit could occur, including sparing cognitive resources that would otherwise be diverted by the demands of listening in difficult environments, reducing the psychosocial sequelae of hearing loss (e.g., social withdrawal and depression), and limiting the interaction between the neurological effects of hearing loss and neuropathology.^2^, ^3^

The Aging and Cognitive Health Evaluation in Elders (ACHIEVE) trial investigated an intervention consisting of HA use and accompanying technologies for cognitive change in older US adults with hearing loss. The trial found little overall effect of the intervention on cognitive change over 3 years.^4^ Our subsequent observational study estimated that 7‐year dementia risk was 33% lower in older adults who received a new HA prescription, compared to those who did not.^5^ Though differences in longitudinal cognitive change were equivocal, effects appeared greatest in those at high risk for cognitive decline, similar to the findings from ACHIEVE.^4^, ^5^, ^6^

Phosphorylated‐tau181 (p‐tau181), amyloid beta (Aβ)42/Aβ40, neurofilament light chain (NfL), and glial fibrillary acidic protein (GFAP) have emerged as key biomarkers of Alzheimer's disease (AD) and related dementias (ADRD). The plasma concentrations of these biomarkers are closely correlated with ADRD pathology^7^ and are prognostic for cognitive decline and dementia.^8^, ^9^ These biomarkers allow for the assessment of treatment effects at an early disease stage (i.e., before clinical endpoints, like dementia, occur) and can provide insight into treatment mechanisms.^10^ Accordingly, we planned to extend our previous study of HA effects on cognition and dementia risk by investigating these biomarkers as outcomes.

The primary aim was to investigate the effect of HA prescription and the frequency of HA use on the mean concentration of plasma biomarkers of ADRD. Additionally, we hypothesized that HA use would influence biomarker concentrations at the upper tail of the distribution, that is, at the level of *marked* neuropathology. This could occur if HA use slowed ADRD progression among individuals who would otherwise develop substantial neuropathology in the absence of intervention. We therefore also aimed to estimate the effect of HA prescription and frequency of HA use on shifting the 90th percentile, rather than the mean, of the biomarker outcome distributions. Finally, we aimed to investigate effect modification by pre‐treatment ADRD risk factors, including the baseline biomarkers, baseline cognition, and apolipoprotein E (*APOE*) ε4 genotype.

## 2 METHODS

### 2.1 Observational data source

**RESEARCH IN CONTEXT**

1. **Systematic review**: Hearing loss is a well‐established dementia risk factor. Prior observational study evidence indicates that treating hearing loss with hearing aids (HAs) could reduce risk of cognitive decline and dementia. We searched MEDLINE and Google Scholar and did not identify previous studies that have investigated the effects of treating hearing loss with HAs on biomarkers of Alzheimer's disease and related dementias (ADRD).
2. **Interpretation**: In our target trial emulation, we found that treating hearing loss with HAs had minimal effects on plasma biomarkers of ADRD in older community‐dwelling individuals in a routine clinical setting.
3. **Future directions**: The putative benefits of hearing aids for dementia prevention may be largely independent of effects on neuropathology. Future long‐term randomized trials or observational studies could examine the impact of sustained HA adherence and consider other treatment mechanisms, such as social isolation, depression, as well as other markers of ADRD pathology.

**FIGURE 1:**
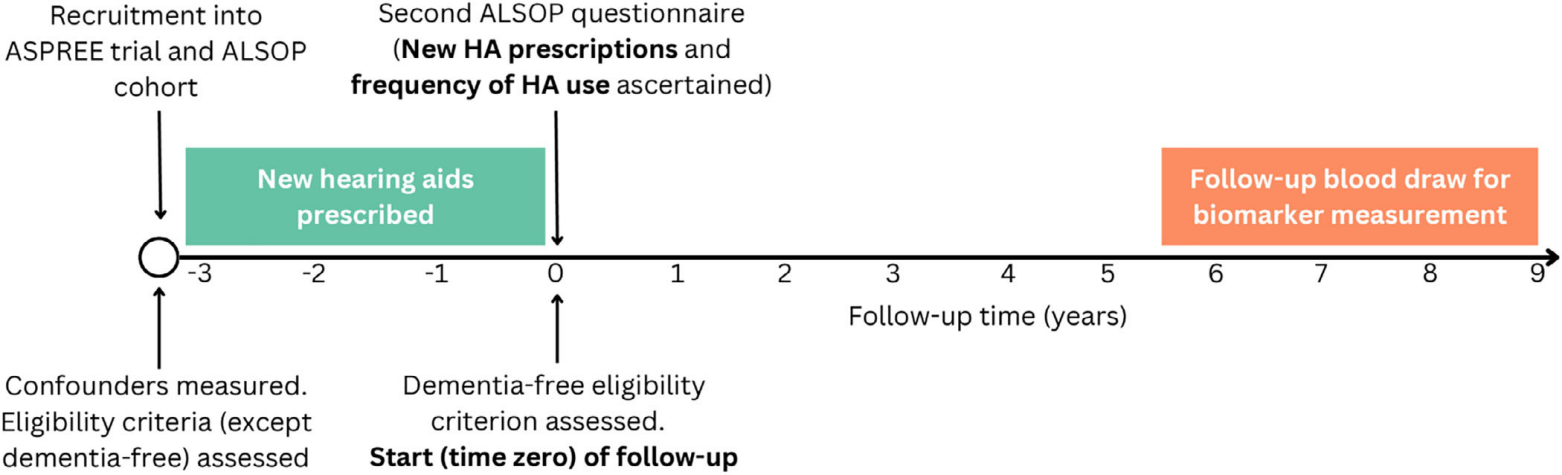
Temporal structure of ASPREE data collection and emulation of each target trial. ALSOP, Australian Longitudinal Study of Older Persons; ASPREE, Aspirin in Reducing Events in the Elderly; HA, hearing aid.

We used observational data from Australian participants of the Aspirin in Reducing Events in the Elderly (ASPREE) study and the Australian Longitudinal Study of Older Persons (ALSOP). ASPREE was a randomized, placebo‐controlled trial investigating low‐dose aspirin for disability‐free survival in 19,114 older individuals in Australia and the United States.^11^, ^12^ In Australia, participants were recruited between 2010 to 2014 aged ≥ 70 years through collaboration with their primary care physician. ASPREE participants were followed prospectively until the trial ended in 2017 and thereafter at annual visits as part of an observational extension (ASPREE‐XT).^13^, ^14^ ALSOP was a substudy in 14,892 Australian ASPREE participants that collected information on medical and social health factors, including hearing function and hearing loss treatment, by questionnaire.^15^ The first ALSOP questionnaire was completed near ASPREE baseline and the second was completed approximately 3 years later (Figure 1).

Data on self‐reported HA prescriptions and frequency of HA use were drawn from the ALSOP surveys. For context, HAs in Australia are provided free of charge to eligible pensioners (aged ≥ 67 years) and to disability health‐care card holders through the Hearing Services Program. Subsidized funding of HAs is also provided by Australia's Department of Veterans’ Affairs and private health insurers. HAs within this study period would have been prescribed by an audiologist within a hearing clinic as part of standard clinical practice.

In ASPREE participants, blood samples were drawn for the measurement of plasma biomarkers of ADRD at baseline (*n* = 11,961) and again approximately 10 years later (*n* = 5243). The protocol for sample collection, processing, and storage has been described in detail.^16^, ^17^ All biomarker assays were completed at the Advanced Research and Diagnostic Laboratory (ARDL) at the University of Minnesota, USA, on the Quanterix HD‐X platform in 2023. The concentrations of Aβ40, Aβ42, NfL, and GFAP were measured on the Simoa Human Neurology 4‐Plex E (N4PE) platform. p‐tau181 was measured using the Simoa pTau181 v2 assay. Inter‐assay and intra‐assay coefficients of variation from a subsample of duplicated measurements were ≤ 5% and < 7% for all biomarkers, respectively.^17^ Although higher performing panels are available, these assays have demonstrated high diagnostic accuracy for AD neuropathology.^18^, ^19^

Finally, we also used data from a subset of ASPREE and ALSOP participants (*n* = 1166) that underwent baseline audiometry assessments as part of the ASPREE Hearing substudy.^20^

### 2.2 Protocols for the target trials and their observational emulation

To guide our observational analysis, we use the target trial framework.^21^, ^22^, ^23^ The first step for using this framework is to define the causal research question by specifying the protocol for the pragmatic randomized trial that would be performed to answer that question (the “target trial”), were it feasible to do so. Next, the observational analysis is designed to “emulate” each protocol component of the target trial, such as its eligibility criteria, as closely as possible. Applying this framework helps to avoid common design‐related biases in observational analyses, such as prevalent user biases.^24^, ^25^

Our analysis is designed to emulate two target trials with different treatment strategies. The protocols for these target trials and their emulation using observational data are described in Table 1. Figure 1 describes the temporal structure of the observational study.

**TABLE 1:** Protocol for the target trials and their observational emulation.

*Protocol component*	*Target trials*	*Observational emulation*
*Eligibility criteria*	Inclusion criteria: Aged 70+ years Moderate or more severe hearing loss (better‐ear 4‐frequency pure tone average ≥ 30 dB HL) 3MS examination score > 77 Community dwelling Exclusion criteria: Dementia Inability/severe difficulty or requiring assistance in performing any activity of daily living Past HA or cochlear implant Contraindication for HAs Unwillingness to wear HAs	Same as target trial, except: Self‐reported hearing problems is used instead of moderate or more severe hearing loss Consent for participation in ASPREE and ALSOP is required No exclusion for HA contraindications or unwillingness to wear HAs Additional ASPREE study exclusion criteria^a^ Eligibility was assessed using variables measured at entry into the ASPREE/ALSOP cohort, except for remaining dementia‐free, which was assessed at time zero (defined as 3 years later; Figure 1).
*Treatment strategies*	*Target trial 1* Use HAs Do not use HAs *Target trial 2* Never use HAs Use HAs rarely/sometimes (> 0 and < 4 times per month) Use HAs often/always (≥ 4 times per month)	Same as target trial.
*Assignment procedure*	Participants are randomly allocated to one of the treatment strategies without blinding.	We classified participants to the treatment groups consistent with their HA prescription/frequency of use data, self‐reported in the second ALSOP questionnaire (Figure 1). As treatment was not randomly assigned, adjustment for baseline confounding is required.
*Outcomes*	Plasma concentrations of p‐tau181, NfL, GFAP, and Aβ42/Aβ40	Same as target trial.
*Follow‐up*	*Starts (time zero)*: At the time of determining eligibility criteria and treatment assignment. *Ends*: At follow‐up blood draw (after 7 years on average), loss to follow‐up, or death.	*Starts (time zero)*: At the third year of the ASPREE study (when the second ALSOP questionnaire is completed, and new HA prescriptions are identified). *Ends*: At follow‐up blood draw (after 7 years on average, i.e., 10 years after recruitment into ASPREE/ALSOP), loss to follow‐up, or death. See Figure 1.
*Causal contrast*	Effect of HA assignment (i.e., intention‐to‐treat effect) in those who survive until biomarker measurement.	In the emulation of the first target trial, the effect of HA prescription. In the emulation of the second target trial, the effect of initiating a given frequency of HA use. All contrasts are conditional on survival.

Abbreviations: 3MS, Modified Mini‐Mental State Examination; Aβ, amyloid beta; ALSOP, Australian Longitudinal Study of Older Persons; *APOE*, apolipoprotein E; ASPREE, Aspirin in Reducing Events in the Elderly; GFAP, glial fibrillary acidic protein; HA, hearing aid; NfL, neurofilament light chain; p‐tau, phosphorylated tau

^a^ Including previous cardiovascular disease events and not being expected to survive for the next 5 years (ASPREE exclusion criteria are described in the primary trial report^12^). See section 2.3 in the main text for identifying assumptions and section 2.4 for statistical analysis.

#### 2.2.1 Eligibility criteria

In the target trials, eligible participants are those who are dementia free, have significant hearing loss (better‐ear 4‐frequency [0.5–4 kHz] pure tone average [PTA] of ≥ 30 dB HL), no past HA use, and do not have contraindications to HAs (e.g., profound/total hearing loss).

The observational emulation applied the same criteria, with two exceptions. First, as audiometric data were only available for a small proportion of the sample, we used presence of self‐reported hearing problems to emulate significant hearing loss. Second, those with contraindications to HAs were not excluded, as these data were unavailable. Participants were assessed for the hearing‐related eligibility criteria at ASPREE/ALSOP baseline and were required to be dementia free at the start (time zero) of follow‐up of the emulation, defined as the third year of the ASPREE study (Figure 1).

#### 2.2.2 Treatment strategies

For the first target trial, the treatment strategies are use HAs (at any frequency) throughout follow‐up and do not use HAs throughout follow‐up. For the second target trial, to explore the dose–response relationship between HA use and the biomarker outcomes, the treatment strategies are never use HAs, use HAs rarely/sometimes (> 0 and < 4 times per month), and use HAs often/always (≥ 4 times per month) throughout follow‐up. The strategies are the same in the observational emulation.

#### 2.2.3 Assignment

In the target trials, participants are randomly allocated to one of the treatment strategies without blinding. In the observational emulation of the first target trial, participants were classified into the “use HAs” strategy if they reported a new HA prescription in the second ALSOP questionnaire (i.e., the third year of the ASPREE study, defined as time zero per above) and to the “do not use HAs” strategy otherwise. In the emulation of the second target trial, participants were assigned to strategies based on their reported frequency of HA use in the second ALSOP questionnaire, with those reporting no HA prescription or no HA use assigned to the “never use HAs” strategy. As the HA treatment was not assigned at random, the observational analysis requires adjustment for baseline confounding (see section 2.3).

#### 2.2.4 Follow‐up

In the target trial, follow‐up begins at treatment assignment and ends at blood collection for biomarker measurement (approximately 7 years later) or death, whichever occurs first. In the observational emulation, follow‐up began at the third year of the ASPREE study, when new HA prescriptions were first reported, and ended in the same manner as the target trial (Figure 1). Loss to follow‐up was defined as missing outcome data not due to death.

#### 2.2.5 Outcomes

Outcomes were the plasma concentrations of p‐tau181, Aβ42/Aβ40, NfL, and GFAP.

#### 2.2.6 Causal contrasts

For the target trials, the primary causal contrast is the effect of HA allocation (irrespective of subsequent adherence, i.e., the intention‐to‐treat effect) on the mean difference scale in survivors. A survival‐conditional effect was the target contrast as estimating such an effect does not require imputation of post‐death outcomes.^26^ The secondary causal contrast is the difference in the 90th percentile (or 10th percentile in the case of Aβ42/Aβ40) of the biomarker distribution between strategies, conditional on survival.

For the observational emulation of the first and second target trials, we used HA prescription and initiation of a given frequency of HA use, respectively, to emulate allocation. All contrasts were conditional on participants being alive when the follow‐up blood draws began.

### 2.3 Identifying assumptions

We assumed that there was no residual confounding after adjusting for baseline covariates including factors associated with hearing aid initiation,^27^, ^28^ self‐rated hearing function, dementia risk factors,^1^ clinical factors, the pre‐treatment plasma ADRD biomarkers, a biomarker‐based dementia risk score (see below), and *APOE* ε4 genotype. See Table S1 in supporting information for all covariates. In all analyses, we additionally adjusted for audiometric hearing loss (see below for missing data handling). Further assumptions, including those concerning missing data, are described in the supporting information (Section S1).

### 2.4 Statistical analysis

Statistical analysis followed a prespecified plan available at https://osf.io/9faew/overview. Analysis was performed using R version 4.3.3.^29^ The analysis code is available at https://github.com/Lachlan‐Cribb/Hearing‐aids‐biomarkers.

#### 2.4.1 Biomarker‐based dementia risk score

Using external data, we created a dementia risk score based on the baseline plasma biomarkers (Section S2 in supporting information). Large scores indicated high‐risk biomarker profiles. We used this risk score to adjust for confounding and assess effect modification by baseline preclinical disease.

#### 2.4.2 Missing data

Missing data are summarized in Table S2 in supporting information. There were missing data in exposures (13%), covariates (mostly < 10%), and the eligibility criteria (< 10%). Audiometric data were missing for most participants (90%), principally due to calendar time as the ASPREE Hearing substudy began during the latter stages of ASPREE recruitment.^20^ The follow‐up ADRD biomarkers were missing for 56% of surviving participants. This proportion of missing outcome data is expected given the approximately 10‐year gap between ASPREE enrolment and the follow‐up blood draw (the mean age of participants was 85 years at this point). The main reasons for missing outcome data were: (1) participants were no longer interested in or able to attend in‐person the ASPREE‐XT visit when the follow‐up blood draw occurred and (2) loss to follow‐up.

We used multiple imputation (MI) to handle all missing data. We chose to use MI rather than restricting the analysis to those with complete data as (1) MI allowed us to incorporate longitudinal auxiliary variables and thereby to satisfy missing data assumptions (see Section S1); (2) MI allowed us to adjust for audiometric hearing in the analyses, despite this only being available for a small proportion of the sample; and (3) MI allowed us to use all available data, including from participants with incomplete records.

MI was implemented using the predictive mean matching method in the R package mice.^30^ Pre‐specified imputation models included outcomes, covariates, treatment, and a subset of longitudinal auxiliary variables (see Table S3 in supporting information). Details of the MI implementation are available in Section S3 in supporting information. We used bootstrapping (250 samples) followed by MI to obtain confidence intervals.^31^ Two imputed datasets were created within each bootstrap sample. Point estimates and confidence interval limits were computed by pooling across imputed datasets and bootstrap samples using established formulas.^31^

#### 2.4.3 Estimation

Mean differences between strategies were estimated using targeted maximum likelihood estimation (TMLE).^32^ The TMLE algorithm begins by fitting models for the conditional mean of the outcome and for the propensity score. Next, the fitted outcome model is “fluctuated” using the estimated propensity score and the g‐computation procedure is applied to the updated outcome predictions. With parametric models, the TMLE estimator is consistent if either the outcome or propensity score model is correctly specified. We used pre‐specified generalized linear models (GLMs) for estimating the conditional mean of the outcome and the propensity score (see Table S1). Due to non‐convergence in some bootstrap samples, product terms that were pre‐specified for inclusion in the treatment model (e.g., between sex and hearing variables) were removed. Propensity scores were truncated at *sqrt*(*n* x ln(*n*)) / 5, where *n* is the sample size.^33^ To estimate effects on the quantile difference scale, we used an inverse probability weighted estimator.^34^, ^35^ We refer to the output of these statistical estimators as “effect estimates,” noting that they admit a causal interpretation only if the above‐described identifying assumptions hold.

To investigate effect modification (on the mean difference scale), we used TMLE to estimate the parameters of a working marginal structural model (see Section S4 in supporting information).^36^

### 2.5 Sensitivity analyses

We conducted sensitivity analyses to examine underpinning assumptions:

1. We applied the same ≥ 30 dB HL hearing loss inclusion criterion as the target trials, using the multiply imputed audiometry data in the full sample.
2. We used skin cancer physical exam in the year preceding time zero as a negative treatment control. Estimates that differ substantially from null may indicate residual confounding (e.g., related to health‐care use).
3. If some biomarker outcome data were missing because of unmeasured neurocognitive decline leading to loss to follow‐up, this could result in bias. To account for this, we conducted two additional analyses using a modified MI approach, known as not‐at‐random fully conditional specification (NARFCS), wherein shift constants of 0.05 and 0.25 standard deviations (−0.05 and −0.25 in the case of Aβ42/Aβ40) were added to each imputed outcome value.^37^, ^38^
4. We used multivariate adaptive regression splines (with interactions of degree 2) to fit the treatment and outcomes models in TMLE, a more flexible and data‐adaptive approach than the primary GLMs.^39^
5. We repeated the primary analysis without adjusting for multiply imputed audiometrically assessed hearing loss.

## 3 RESULTS

A flow diagram describing participant selection is presented in Figure S1 in supporting information. Across imputed datasets, the median (Q1, Q3) sample size of eligible participants for each target trial emulation was 2842 (2808, 2872). A median of 735 (711, 756) participants received a new HA prescription by the start of follow‐up. Characteristics of the sample are described in Table 2. The median age was 74 years and 48% were female. The median interval between time zero and the follow‐up blood draw was 7.3 years (6.5, 8.1). The follow‐up blood draw was precluded by death for a median of 89 participants (12%) who received a new HA prescription and for 209 participants (10%) who did not. The distributions of the biomarker outcomes are displayed in Figure S2 in supporting information. In participants with audiometry data available, the median hearing loss was 29 dB HL. Most (81%) had ≥ 20 dB HL.

**TABLE 2:** Sample characteristics^*^ ^.^

*Characteristic*	*Summary*	
	*New HA prescription*	*No new HA prescription*
*N*	735 (711, 756)	2107 (2078, 2137)
Age	74 (72, 78)	73 (71, 76)
Woman	360 (49%)	1014 (48%)
White race	720 (98%)	2087 (99%)
Education		
< 9 years	124 (17%)	282 (13%)
9–11 years	226 (31%)	657 (31%)
12 years	86 (12%)	238 (11%)
13–15 years	108 (15%)	328 (16%)
16 years	59 (8%)	179 (9%)
17–21 years	130 (18%)	422 (20%)
3MS overall score	95 (91, 97)	95 (92, 97)
BMI, median (IQR)	27 (25, 30)	27 (25, 30)
History of diabetes	62 (9%)	144 (7%)
Antihypertensive medication use	25 (3%)	61 (3%)
Smoking status		
Never	409 (56%)	1142 (54%)
Former	306 (42%)	914 (43%)
Current	18 (2%)	51 (2%)
4‐frequency PTA in better ear (dB HL)	33 (26, 40)	27 (20, 33)
Systolic blood pressure	139 (128, 151)	139 (127, 150)
*APOE* ε4 positivity	179 (24%)	549 (26%)
*Pre‐treatment biomarkers*		
Risk score (%)^†^	6 (3, 10)	5 (3, 10)
p‐tau181 (pg/mL)	32 (23, 44)	32 (24, 44)
Aβ42/Aβ40 x 1000	61 (54, 69)	61 (54, 69)
GFAP (pg/mL)	119 (89, 161)	116 (85, 160)
NFL (pg/mL)	20 (15, 25)	19 (15, 25)

Abbreviations: 3MS, Modified Mini‐Mental State Examination; Aβ, amyloid beta; ALSOP, Australian Longitudinal Study of Older Persons; *APOE*, apolipoprotein E; ASPREE, Aspirin in Reducing Events in the Elderly; BMI, body mass index; GFAP, glial fibrillary acidic protein; HA, hearing aid; HL, hearing loss; IQR, interquartile range; NfL, neurofilament light chain; PTA, pure tone average; p‐tau, phosphorylated tau.

^*^ Measured at recruitment into ASPREE/ALSOP study (3 years preceding time zero of follow‐up). Summary statistics are median (Q1, Q3) for numeric variables and *n* (%) for categorical variables, averaged over the imputed datasets.

^†^ Dementia risk score estimated based on plasma biomarkers in out‐of‐sample data (see Section S2 in supporting information).

### 3.1 Estimated effects on mean difference scale

The estimated mean differences between strategies among survivors are displayed in Table 3. All estimates were close to null. For example, in the emulation of the first target trial, the estimated mean concentration of p‐tau181 was 1.8 pg/mL (95% confidence interval [CI]: −0.6, 4.1) greater under HA prescription than under no HA prescription. For the emulation of the second target trial, the estimated mean concentration of p‐tau181 was 2.3 pg/mL (−1.0, 5.5) greater under initiation of always using HAs compared to never using HAs.

**TABLE 3:** Estimate effects on mean difference scale.

Biomarker & strategy	Estimated mean	Estimated mean difference (95% CI)
**First target trial**		
*p‐tau181 (pg/mL)*		
No HA prescription	35.8	Reference
HA prescription	37.6	1.8 (−0.6, 4.1)
*Aβ42/Aβ40 x 1000*		
No HA prescription	61.5	Reference
HA prescription	60.8	−0.7 (−2.6, 1.2)
*GFAP (pg/mL)*		
No HA prescription	175.0	Reference
HA prescription	172.8	−2.2 (−14.5, 10.1)
*NfL (pg/mL)*		
No HA prescription	31.3	Reference
HA prescription	31.4	0.1 (−7.8, 8.0)
**Second target trial**		
*p‐tau181 (pg/mL)*		
No HA initiation	35.8	Reference
Initiate using HAs rarely/sometimes	37.5	1.7 (−0.9, 4.3)
Initiate using HAs often/always	38.1	2.3 (−1.0, 5.5)
*Aβ42/Aβ40 x 1000*		
No HA initiation	61.5	Reference
Initiate using HAs rarely/sometimes	61.5	−0.0 (−2.4, 2.4)
Initiate using HAs often/always	60.3	−1.2 (−3.4, 1.0)
*GFAP (pg/mL)*		
No HA initiation	174.1	Reference
Initiate using HAs rarely/sometimes	177.0	2.9 (−7.0, 12.7)
Initiate using HAs often/always	173.0	−1.1 (−12.0, 9.8)
*NfL (pg/mL)*		
No HA initiation	31.1	Reference
Initiate using HAs rarely/sometimes	32.6	1.5 (−0.1, 3.9)
Initiate using HAs often/always	31.2	−0.0 (−2.1, 2.2)

Abbreviations: Aβ, amyloid beta; CI, confidence interval; GFAP, glial fibrillary acidic protein; HA, hearing aid; NfL, neurofilament light chain; p‐tau, phosphorylated tau.

### 3.2 Estimated effects on quantile difference scale

Estimates on the quantile difference scale among survivors are displayed in Table 4. All estimates were close to null. For example, in the emulation of the first target trial, the estimated 90th percentile of p‐tau181 was 3.3 pg/mL (−2.3, 8.9) greater under HA prescription than under no prescription.

**TABLE 4:** Estimated effects on quantile difference scale.

Biomarker & strategy	Estimated 90th percentile (10th percentile for Aβ42/Aβ40)	Quantile difference (95% CI)
**First target trial**		
*p‐tau181 (pg/mL)*		
No HA prescription	55.2	Reference
HA prescription	58.5	3.3 (−2.3, 8.9)
*Aβ42/Aβ40 x 1000*		
No HA prescription	43.8	Reference
HA prescription	40.5	−3.3 (−7.1, 0.5)
*GFAP (pg/mL)*		
No HA prescription	279.2	Reference
HA prescription	274.5	−4.7 (−29.1, 19.7)
*NfL (pg/mL)*		
No HA prescription	49.2	Reference
HA prescription	50.7	1.5 (−4.2, 7.3)
		
**Second target trial**		
*p‐tau181 (pg/mL)*		
No HA initiation	55.4	Reference
Initiate using HAs rarely/sometimes	57.2	1.9 (−4.3, 8.0)
Initiate using HAs often/always	59.4	4.1 (−2.5, 10.7)
*Aβ42/Aβ40 x 1000*		
No HA initiation	43.8	Reference
Initiate using HAs rarely/sometimes	43.7	−0.2 (−4.1, 3.7)
Initiate using HAs often/always	39.7	−4.2 (−8.1, −0.3)
*GFAP (pg/mL)*		
No HA initiation	279.1	Reference
Initiate using HAs rarely/sometimes	278.3	−0.8 (−25.6, 24.1)
Initiate using HAs often/always	271.4	−7.7 (−30.7, 15.3)
*NfL (pg/mL)*		
No HA initiation	49.0	Reference
Initiate using HAs rarely/sometimes	51.1	2.1 (−5.9, 10.1)
Initiate using HAs often/always	50.7	1.7 (−4.9, 8.4)

Abbreviations: Aβ, amyloid beta; CI, confidence interval; GFAP, glial fibrillary acidic protein; HA, hearing aid; NfL, neurofilament light chain; p‐tau, phosphorylated tau.

### 3.3 Effect modification

**FIGURE 2:**
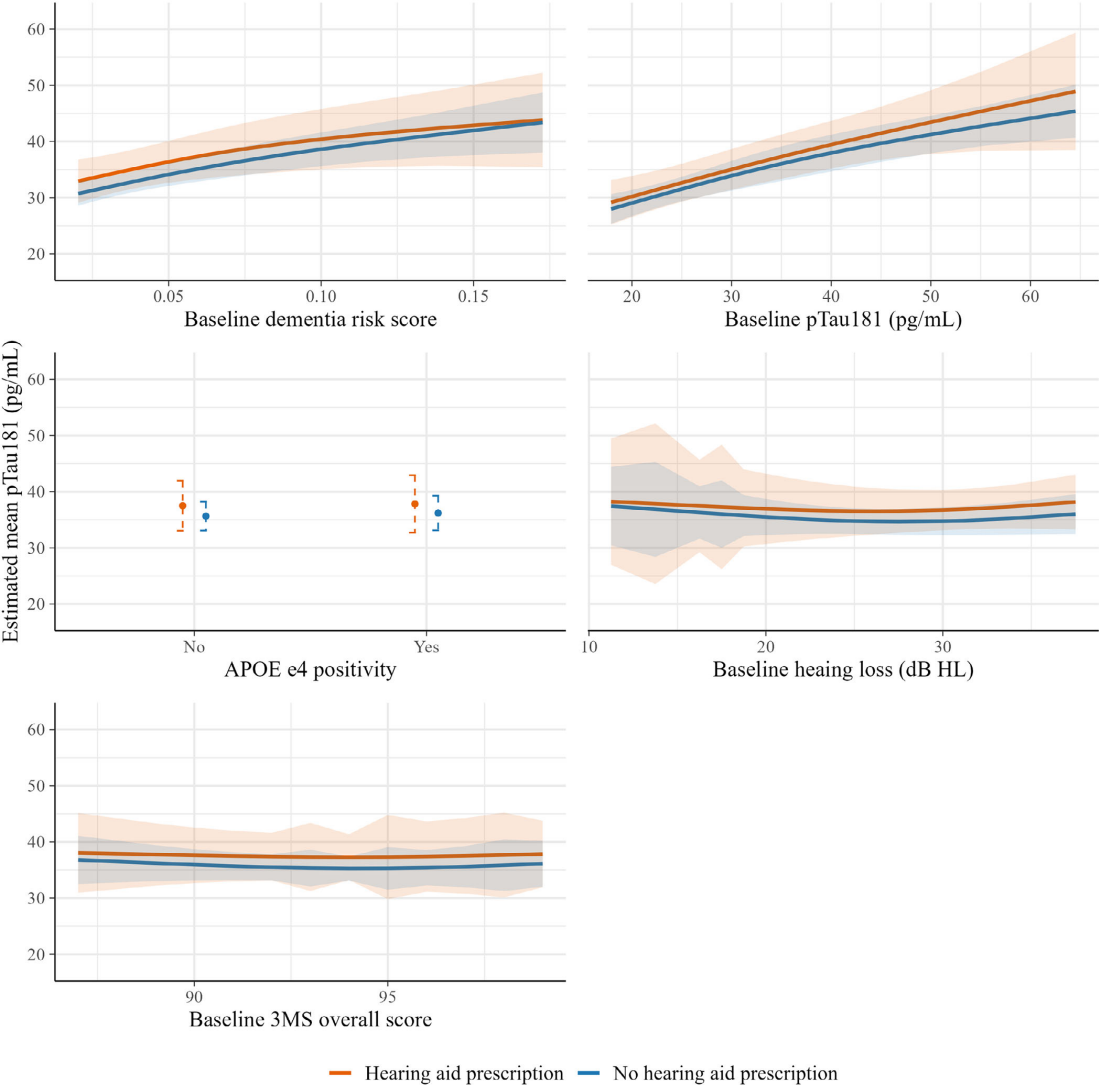
Estimated effect of HA prescription on p‐tau181 in survivors, by effect modifiers. For continuous effect modifiers, the *x* axis ranges from the 2.5th to the 97.5th percentiles of the effect modifier. Ribbons are bootstrap 95% confidence intervals. 3MS, Modified Mini‐Mental State Examination; *APOE*, apolipoprotein E; HA, hearing aid; HL, hearing loss; p‐tau, phosphorylated tau.

The estimated effects of HA prescription did not substantially differ across the levels of the baseline effect modifiers. For example, in those with relatively poor baseline cognition (Modified Mini‐Mental State Examination [3MS] overall score of 85), the estimated mean concentration of p‐tau181 was 0.9 pg/mL greater under HA prescription than under no HA prescription. In those with good baseline cognition (3MS overall score of 100), the estimated mean difference was 1.6 pg/mL (Figure 2). Findings were similar for the other biomarker outcomes (Figures S3–S5 in supporting information).

### 3.4 Sensitivity analyses

Estimated effects of the skin cancer physical exam negative treatment control were close to null for all biomarker outcomes (Table S4 in supporting information). Neither restricting the sample to those with hearing loss estimated as ≥ 30 dB (based on the multiply imputed audiometry data), delta adjustments to the imputed outcome data, excluding audiometrically assessed hearing loss from the covariate set, nor using multivariate adaptive regression splines to fit the treatment and outcome models substantively altered the results (Table S5–S8 in supporting information).

## 4 DISCUSSION

Our observational analysis found that HA prescription and the initiation of different self‐reported frequencies of HA use had minimal association with plasma biomarkers of ADRD measured after approximately 7 years of follow‐up. Counter to our supposition that treatment effects may be greater among those with elevated pre‐treatment ADRD risk, we found that effect estimates differed little by baseline cognition or baseline biomarker concentrations. Finally, we found that the estimated effects of HA prescription and frequency of HA use on the 90th (10th for Aβ42/Aβ40) percentiles of the biomarker distributions were similarly minimal.

There are several plausible explanations for our findings. A first is that there is truly no effect of treating hearing loss with HAs on the aspects of ADRD pathology that are reflected in the studied biomarkers. Instead, the putative benefits of HAs for delaying or preventing dementia may work through other mechanisms, such as reducing social isolation (an establishment risk factor for dementia), enhancing cognitive reserve through cognitive engagement/stimulation, or via neurological pathways not captured by the biomarkers studied here (e.g., neuroinflammatory).^3^ A second explanation is that, though there could be a real effect of HAs on ADRD pathology, it may only be detectable with high and sustained HA adherence (which does not reflect the routine clinical setting^40^, ^41^). Finally, the effect of treating hearing loss with HAs in this older (aged ≥ 70 years) population may differ from the effect of initiating the treatment at a younger age. Indeed, midlife appears to be a particularly crucial period and interventions initiated at this time may have the greatest benefit.^1^

Though an increasing number of studies are considering the use of plasma biomarkers as dementia surrogate endpoints,^42^ we are not aware of previous studies that have investigated HAs and ADRD biomarkers. Several studies have investigated the association between hearing loss itself and biomarkers of ADRD. Though the overall picture is ambiguous, these tend to suggest that the cerebrospinal fluid (CSF) concentrations of p‐tau and total tau are modestly elevated in those with hearing loss, but that there is little association between hearing loss and levels of CSF or positron emission tomography Aβ.^43^, ^44^, ^45^

Strengths of the study include the long duration of follow‐up between HA prescription and biomarker measurement and the use of a study design approach that helps to avoid common biases.^25^ Limitations include the use of self‐reported hearing problems to emulate ≥ 30 dB HL hearing loss in the primary analysis, an imperfect surrogate.^46^ The hearing loss level for approximately half of the sample was below this 30 dB HL level, though most participants had some degree of loss. We also had no information on participants with contraindications for HAs, though we expect this proportion would be small. Second, inclusion in the observational sample required that participants met the eligibility criteria for, and consented to participate in, the ASPREE trial. At baseline, Australian participants of the ASPREE trial tended to be healthier than the age‐matched general Australian population, with a lower prevalence of some chronic diseases, like diabetes.^47^ The sample data therefore differ from the target population (as specified by the eligibility criteria of the target trials) in physical health characteristics, a source of bias if these physical health factors are effect modifiers. Third, the timing of confounder measurement was not perfectly aligned with the time of HA prescription. Residual confounding could result if confounders changed substantially between the time of their measurement at ASPREE/ALSOP baseline and the time of HA prescription (up to 3 years later).^25^

A fourth limitation is the missing data, especially in audiometric hearing loss and the biomarker outcomes. Though we used multiple imputation to handle this missing data, the validity of our inferences is sensitive to violations of missing data assumptions. Finally, as we did not have longitudinal data on HA adherence, we were restricted to estimating the effect of HA prescription or initiation. Though these effects, analogous to the intention‐to‐treat effects in a trial, are relevant to the real‐world clinical setting (in which HA non‐adherence is common^40^, ^41^), it would have been additionally valuable to estimate the effect of sustained HA adherence.

In conclusion, our study found minimal association between HA prescription or HA use and ADRD biomarkers after a 7‐year follow‐up. Future long‐term randomized trials or observational studies could examine the impact of sustained HA adherence and consider other markers of ADRD pathology.

## CONFLICT OF INTEREST STATEMENT

Dr. R.C. Shah reports being the site principal investigator or sub‐investigator for AD clinical trials for which his institution (Rush University Medical Center) is compensated (Athira Pharma, Inc., Annovis Bio, Inc., Edgewater NEXT, Eisai, Inc., and Genentech, Inc.). Dr. Shah also reports a compensated scientific advisory board agreement with Lundbeck, Inc., and a non‐compensated consultant agreement with Novo Nordisk regarding diagnosis and management of dementia. Dr T. T.‐J. Chong reports receiving honoraria for lectures from Roche. None of these are directly related to the work in this manuscript. All other authors report no disclosures. Author disclosures are available in the Supporting Information.

## CONSENT STATEMENT

ASPREE and ASPREE‐XT were approved by the ethics review board at each participating institution. They were performed in accordance with the principles of the Declaration of Helsinki. All participants provided written informed consent.

## Supporting information



Supporting Information

Supporting Information

Supporting Information

Supporting Information

Supporting Information

Supporting Information

Supporting Information

Supporting Information

Supporting Information

Supporting Information

Supporting Information

Supporting Information

Supporting Information

Supporting Information

Supporting Information

## Data Availability

Data from the ASPREE study and associated substudies is available to researchers who receive approval for an expression of interest in the ASPREE access management system (https://ams.aspree.org/public/request‐data/access‐aspree‐data/).
