# Supplementary material for: The effect of treating hearing loss with hearing aids on plasma biomarkers of Alzheimer's disease and related dementias
Source: Alzheimers Dement (Amst). 2026 Jun 23;18(2):e70397. doi: 10.1002/dad2.70397 (PMC13290640; doi:10.1002/dad2.70397)
Supplement: Supplementary file 1 — Supporting Information [file DAD2-18-e70397-s003.docx]

**SUPPLEMENTARY MATERIAL FOR:**

**The Effect of Treating Hearing Loss with Hearing Aids on Plasma Biomarkers of Alzheimer’s Disease and Related Dementias**

*Contents*

[1. Identifying assumptions 2](#_Toc224385787)

[2. Constructing the biomarker-based dementia risk score 5](#_Toc224385788)

[3. Multiple imputation 6](#_Toc224385789)

[4. TMLE estimation of marginal structural model 6](#_Toc224385790)

[References 7](#_Toc224385791)

### 1. Identifying assumptions

The primary causal estimand, for the first emulated target trial, is the effect of hearing aid (HA) prescription versus no HA prescription among survivors. In potential outcomes notation, we define this as $E\left[ Y^{a=1,m_{Y}=0}|D=0 \right]-E\left[ Y^{a=0,m_{Y}=0}|D=0 \right]$, with $a=1$ representing HA prescription and $a=0$ no prescription, $M_{Y}$ representing an indicator of missing outcome data ($M_{Y}=0$ if outcome not missing), and *D* representing a death indicator (with $D=0$ indicating survival). That is, the causal estimand is the mean difference in the concentration of a given biomarker in survivors under HA prescription and no HA prescription and the absence of missing outcome data. This section describes the assumptions required to identify this causal estimand.

#### 1.1 Missingness directed acyclic graph


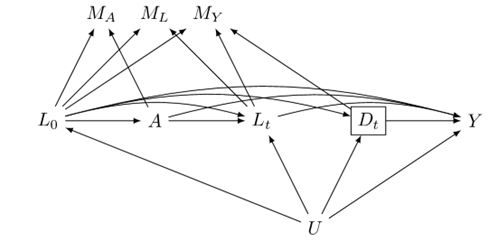


The above figure displays a missingness directed acyclic graph (m-DAG) [1] for a biomarker outcome *Y*. *L_0_* is a vector of baseline confounders, *A* is treatment, $L_{t}$ is a vector of covariates at time *t*, $D_{t}$ is death by time *t*, *U* is a vector of unmeasured variables, and $M_{L}$, *M_A_*, and *M_Y_* are indicators for missingness in the covariates, treatment, and outcome, respectively. The rectangle around $D_{t}$ represents conditioning (by restriction to survivors).

Given the presence of missing data, additional assumptions beyond the standard assumptions of exchangeability, positivity, and consistency, are required for our estimand to be “recoverable”, i.e., estimable without bias from the observed data alone. To examine these missing data assumptions, we make reference to recent theoretical and empirical recoverability results for a set of “canonical” m-DAGs [1,2]. A key empirical finding from this work is that, even for theoretically non-recoverable estimands, appropriate multiple imputation approaches can yield approximately unbiased causal effect estimates, so long as the outcome is conditionally independent of its own missingness [2]. This latter condition holds for our m-DAG as we have that $Y\perp M_{Y}|L_{0},{A,L}_{t},D=0$. Note that this conditional independence would not hold without including the longitudinal auxiliary variables $L_{t}$ in the conditioning set. In the next paragraphs, we discuss the other assumptions required for identification.

*1.2 Conditional exchangeability*

Conditional exchangeability refers to the independence of potential outcomes from the treatment received, given the adjustment variables. For HA treatment, we assume that conditional exchangeability holds given the baseline confounders and survival until biomarker measurement, i.e., that $Y^{a,m_{Y}=0}\perp A|L_{0},D=0$ for all *a*. This assumption is represented in the m-DAG by the absence of unblocked backdoor paths from the outcome to the HA treatment variable after conditioning on these two nodes. Note that, in the m-DAG, there are no direct or indirect paths from treatment to death. Consequently, there are no unblocked backdoor paths created by restricting the analysis to those who survive until outcome measurement. We previously found minimal association between HA use and overall mortality over 7 years and thus believe this assumption is plausible [3].

#### 1.3 Positivity

Positivity holds when all participant subgroups within the target population have some positive probability of receiving each treatment level. For this study, we require positivity for missingness and for HA treatment. For missingness, we assume that for all $\left( l_{0},a,l_{t} \right)$ such that $f_{L_{0},A,L_{t},D}\left( l_{0},a,l_{t},0 \right)\neq0$, $\Pr\left[ M=0 | L_{0}=l_{0},A=a,L_{t}=l_{t},D=0 \right]$ > 0 in the target population. I.e., that there are some surviving individuals with non-missing data for every subgroup defined by values of the treatment, baseline covariates, and the auxiliary variables. For HA treatment, we assume that if $f_{L_{0},D}\left( l_{0},0 \right)\neq0$ then $\Pr\left[ A=a | L_{0}=l_{0}, D=0 \right]$ > 0 for all *l_0_* and all *a* among survivors.

#### 1.4 Consistency

Consistency refers to the potential outcomes under an intervention being equal to the observed outcomes for those who received that intervention*.* I.e., that $Y^{a,m_{Y}=0}=Y$ for those with *A* = *a* and $M_{Y}=0$. In our setting, there are plausibly multiple versions of HA prescription (e.g., behind-the-ear vs in-the-ear HAs) that could result in different biomarker outcomes. Nevertheless, under assumptions that we expect to hold here (i.e., the absence of unmeasured common causes of HA prescription and the version of HA prescription received), our estimand remains estimable and has a natural interpretation: the average effect of HA prescription on the outcome with the versions of HA prescription drawn from the distribution of versions of HA prescription in the target population [4].

#### 1.5 Measurement error

We assume that HA prescription is measured without error. The biomarker outcomes, because they are measured from plasma and provide only a proxy measure of their CNS concentration, are measured with some error. We assume that this measurement error does not differ by (i.e., is non-differential with respect to) the HA exposures. This is plausible as there is no clear mechanism linking the technical and biological noise in the biomarker measurement with an individual’s hearing treatment. We therefore do not expect the outcome measurement error to introduce meaningful bias for our analysis, as independent and non-differential error in a continuous outcome does not bias mean differences [5].

Other variables, including the baseline covariates, are likely measured with some error. This is a limitation that could lead to some residual confounding. The frequency of HA use is also likely measured with error, as self-report tends to systematically overestimate actual HA use frequency [6]. Under the assumption that the latter error does not differ by the biomarker outcomes (after accounting for the baseline covariates, including baseline cognition), we would expect the measurement error in HA use to introduce bias towards the null [5].

### 2. Constructing the biomarker-based dementia risk score

To create the all-cause dementia risk score based on the pre-treatment biomarkers, we used the following algorithm:

1. We created an “external” dataset that contained ASPREE study participants that did not meet the eligibility criteria for this study and who had baseline biomarker data available (n = 8,329 individuals, 785 deaths, and 615 dementia cases).
2. In the external dataset, we create a person-period (discrete time survival) dataset that included, for each participant, as many rows of data as they had 2-year follow-up intervals, ending either at dementia, death, loss to follow-up, or after 10 years
3. In the external dataset, we fitted SuperLearner [7] models for the discrete time of hazard of dementia and of death, including time and the biomarkers as predictors
4. In the main analysis dataset, we computed the estimated 10-year cumulative incidence of dementia (the risk score) as a function of the hazards from the fitted models in (3)

The use of external data for fitting the models for the risk score prevents bias in the use of the risk score related to overfitting [8]. Learners included in the SuperLearner for estimating the discrete time hazards were GLMs, generalised additive models, multivariate adaptive regression splines, Bayesian adaptive regression trees, and gradient boosting. All algorithms used default hyperparameters of the SuperLearner R package, except for gradient boosting (minimum size of leaf node set to 25 and maximum tree depth set to 3). 10-fold cross-validation was used to estimate the ensemble weights [9]. Random forests were pre-specified for inclusion but were removed due to computational limitations.

### 3. Multiple imputation

To ensure compatibility with the main analysis method (described below), non-linear and two-way product terms for key variables were included [10]. The subsets of auxiliary variables, non-linear and product terms (excluding those between treatment and pre-specified effect-modifiers, which were always included) to include in imputation models were determined using LASSO variable selection [11]. In the procedure, quadratic terms and product terms were passively imputed [10]. MI was performed once to impute missing data for all analyses (primary and sensitivity).

### 4. TMLE estimation of marginal structural model

To investigate modification of the effect of HA prescription on the mean difference scale, we used TMLE to estimate the parameters of a working marginal structural model. This model included a term for the treatment, the effect modifier (modelled with a restricted cubic spline with knots at the 10^th^, 50^th^, and 90^th^ percentiles for continuous effect modifiers), and their product(s). We then plotted the estimated outcome mean under each treatment strategy, across levels of the effect modifiers.

### References

[1] Moreno-Betancur M, Lee KJ, Leacy FP, White IR, Simpson JA, Carlin JB. Canonical causal diagrams to guide the treatment of missing data in epidemiologic studies. Am J Epidemiol 2018;187:2705–15.

[2] Zhang J, Dashti SG, Carlin JB, Lee KJ, Moreno-Betancur M. Recoverability and estimation of causal effects under typical multivariable missingness mechanisms. Biom J 2024;66:2200326. https://doi.org/10.1002/bimj.202200326.

[3] Cribb L, Moreno-Betancur M, Pase MP, Wolfe R, Britt CJ, Zhou Z, et al. Treating Hearing Loss with Hearing Aids for the Prevention of Dementia and Cognitive Decline. Neurology n.d.;(in press).

[4] VanderWeele TJ, Hernán MA. Causal Inference Under Multiple Versions of Treatment. J Causal Inference 2013;1:1–20. https://doi.org/10.1515/jci-2012-0002.

[5] Lash TL, VanderWeele TJ, Haneause S, Rothman K. Modern Epidemiology. Philadelphia, UNITED STATES: Wolters Kluwer Health; 2021.

[6] Laplante-Lévesque A, Nielsen C, Jensen LD, Naylor G. Patterns of Hearing Aid Usage Predict Hearing Aid Use Amount (Data Logged and Self-Reported) and Overreport. J Am Acad Audiol 2020;25:187–98. https://doi.org/10.3766/jaaa.25.2.7.

[7] Laan MJ van der, Polley EC, Hubbard AE. Super Learner. Stat Appl Genet Mol Biol 2007;6. https://doi.org/10.2202/1544-6115.1309.

[8] Abadie A, Chingos MM, West MR. Endogenous Stratification in Randomized Experiments. Rev Econ Stat 2018;100:567–80. https://doi.org/10.1162/rest_a_00732.

[9] Phillips RV, van der Laan MJ, Lee H, Gruber S. Practical considerations for specifying a super learner. Int J Epidemiol 2023;52:1276–85. https://doi.org/10.1093/ije/dyad023.

[10] Dashti SG, Lee KJ, Simpson JA, White IR, Carlin JB, Moreno-Betancur M. Handling missing data when estimating causal effects with Targeted Maximum Likelihood Estimation. Am J Epidemiol 2024:kwae012. https://doi.org/10.1093/aje/kwae012.

[11] A comparison of strategies for selecting auxiliary variables for multiple imputation - Mainzer - 2024 - Biometrical Journal - Wiley Online Library n.d. https://onlinelibrary.wiley.com/doi/full/10.1002/bimj.202200291 (accessed November 19, 2024).
