# Supplementary material for: The effect of treating hearing loss with hearing aids on plasma biomarkers of Alzheimer's disease and related dementias
Source: Alzheimers Dement (Amst). 2026 Jun 23;18(2):e70397. doi: 10.1002/dad2.70397 (PMC13290640; doi:10.1002/dad2.70397)
Supplement: Supplementary file 2 — Supporting Information [file DAD2-18-e70397-s004.docx]

### **Figure A1. Flow diagram**

Australian participants of ASPREE trial (n=16,703)

Participated in ALSOP cohort (n=14,908)

Completed follow-up blood draw (n=845)*

Completed follow-up blood draw (n=268)*

- Prior treatment for hearing loss (n=4,206)*
- Death/dementia before ALSOP year 3 (n=405)
- No self-reported hearing problems (n=7,455)*

Survived until time of follow-up blood draw (n=1,896)*

No hearing aid prescription reported in ALSOP year 3 (n=2,107)*

Survived until time of follow-up blood draw (n=645)*

New hearing aid prescription reported in ALSOP year 3 (n=735)*

Included (n=2,842)*

* Median across imputed datasets
