# Supplementary material for: The effect of treating hearing loss with hearing aids on plasma biomarkers of Alzheimer's disease and related dementias
Source: Alzheimers Dement (Amst). 2026 Jun 23;18(2):e70397. doi: 10.1002/dad2.70397 (PMC13290640; doi:10.1002/dad2.70397)
Supplement: Supplementary file 3 — Supporting Information [file DAD2-18-e70397-s001.docx]

### **Figure A2. Density of biomarker outcomes by new hearing aid prescription**


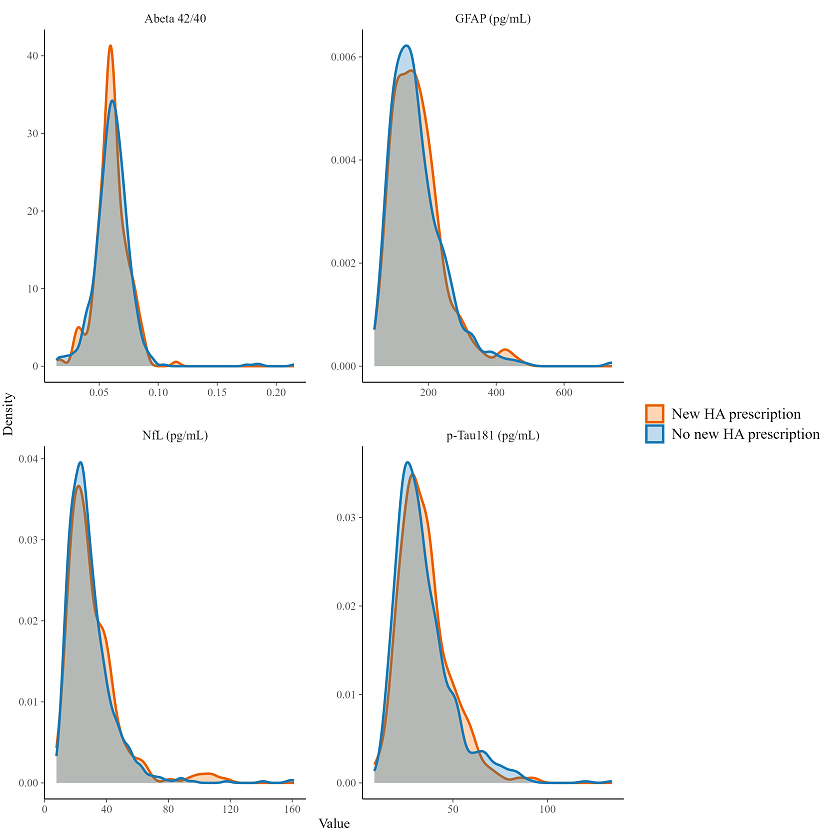


Density plots are obtained from eligible sample with complete exposure and outcome data (n = 999) and are not adjusted for confounding. For visual clarity, outcomes are truncated at 0.1 and 99.9 percentiles.
