# Supplementary material for: The effect of treating hearing loss with hearing aids on plasma biomarkers of Alzheimer's disease and related dementias
Source: Alzheimers Dement (Amst). 2026 Jun 23;18(2):e70397. doi: 10.1002/dad2.70397 (PMC13290640; doi:10.1002/dad2.70397)

### **Figure A4. Estimated effect of HA prescription on GFAP on mean difference scale in survivors, by effect modifiers**


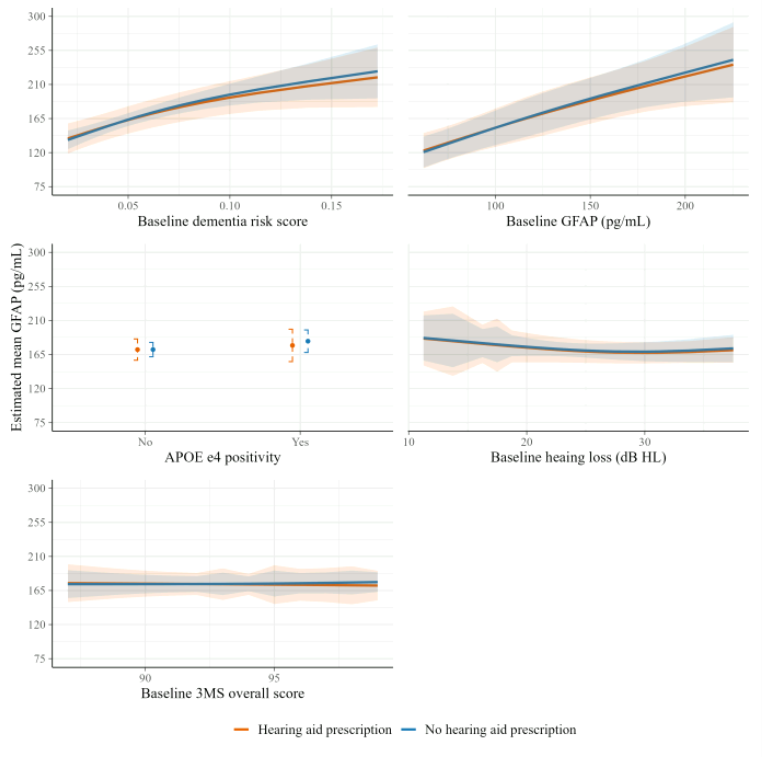

Supplement: Supplementary file 5 — Supporting Information [file DAD2-18-e70397-s014.docx]
