# Supplementary material for: The effect of treating hearing loss with hearing aids on plasma biomarkers of Alzheimer's disease and related dementias
Source: Alzheimers Dement (Amst). 2026 Jun 23;18(2):e70397. doi: 10.1002/dad2.70397 (PMC13290640; doi:10.1002/dad2.70397)
Supplement: Supplementary file 7 — Supporting Information [file DAD2-18-e70397-s002.docx]

### **Table A1: Prespecified baseline confounders and model specification**

| **Confounder** | **Available ASPREE or ALSOP measures** | **Functional form** | **Product terms in outcome models** |
| --- | --- | --- | --- |
| Time from baseline* | Time between start of follow-up and biomarker measurement | RCS(3) | Treatment |
| Biomarkers of neurodegeneration | pTau-181, NfL, GFAP, Aβ42 / Aβ40, risk score | RCS(3) | Treatment, key covariates^†^, other baseline biomarkers, time from baseline |
| Age | Age | RCS(3) | Treatment, key covariates^†^, baseline biomarkers |
| Gender | Gender | Binary | Treatment, key covariates^†^, baseline biomarkers |
| BMI | BMI | RCS(3) | - |
| Education | Years of education | RCS(3) | Treatment, key covariates^†^, baseline biomarkers |
| Race | Race | Categorical | - |
| Socioeconomic status | Postcode based metric of socioeconomic status (Index of Relative Socio-economic Advantage and Disadvantage) | RCS(3) | - |
|  | Income (4-point scale ranging from <20,000 AUD to >100,000 AUD) | Quadratic | - |
| Social isolation | Sum score of social engagement questionnaire items | RCS(3) | - |
| Physical activity | Physical activity (self-rated; 4-point scale representing physical activity performed in a typical week, ranging from 0 [rarely/never perform physical activity] to 3 [regular vigorous physical activity]) | Quadratic | - |
| Sleep duration | Hours of nightly sleep (self-rated; 6-point scale ranging from <4 hrs to >12hrs) | Quadratic |  |
| Smoking | Smoking status (“current”, “former”, “never”) | Categorical | - |
| Alcohol intake | Typical alcoholic drinks/week | RCS(3) | - |
| Hearing function | Hearing deterioration over last 5 years (self-rated) | Binary | - |
|  | Tinnitus (self-rated; 5-point scale from “never” to “always”) | Quadratic |  |
|  | Difficulty hearing in quiet room (self-rated; 4-point scale from “not at all” to “a lot”) | Quadratic | - |
|  | Difficulty hearing in crowded room (self-rated; 4-point scale from “not at all” to “a lot”) | Quadratic | Treatment |
|  | Pure tone average (PTA) of air conduction thresholds at 0.5, 1, 2, and 4 kHz in the better ear | RCS(3) | Treatment, key covariates^†^, baseline biomarkers |
| Physical health | Physical component score of Short Form-12 | RCS(3) | - |
| Mental health | Mental component score of Short Form-12 | RCS(3) | - |
| Depression | Center for Epidemiological Studies – Depression total score | RCS(3) | - |
| Polypharmacy | Total reported medications | RCS(3) | - |
| History of cancer | Self-reported history of cancer | Binary | - |
| History of diabetes | Self-reported history of diabetes | Binary | - |
| Systolic blood pressure | Systolic blood pressure | RCS(3) | - |
| Cognitive function | 3MS overall score | RCS(3) | Treatment, key covariates^†^, baseline biomarkers |
|  | HVLT-R delayed recall | RCS(3) | Treatment |
| APOE-e4 genotype | APOE-e4 genotype | Linear | Treatment, key covariates^†^, baseline biomarkers |
| Frailty | Deficit-Accumulation Frailty Index | RCS(3) | Treatment, key covariates^†^, baseline biomarkers |
| Visual impairment | 6-point self-rated eyesight scale, with responses ranging from “excellent” to “completely blind” | Quadratic | - |
| Chronic kidney disease | Chronic kidney disease |  | - |
| eGFR | eGFR | RCS(3) | - |
| Liver function | First two principal components of available liver function test results (e.g., AST, ALT, ALP, Bilirubin, GGT) | RCS(3) | - |

Confounders are measured at recruitment into ASPREE or ALSOP studies. * Outcome model only. RCS(3) = restricted cubic spline with knots at the 10^th^, 50^th^, and 90^th^ percentiles. ^†^ Age, gender, education, 3MS overall score, APOE e4 genotype, frailty. Product terms were pre-specified for inclusion in treatment models but were excluded due to model non-convergence in some bootstrap samples.
