# Supplementary material for: The effect of treating hearing loss with hearing aids on plasma biomarkers of Alzheimer's disease and related dementias
Source: Alzheimers Dement (Amst). 2026 Jun 23;18(2):e70397. doi: 10.1002/dad2.70397 (PMC13290640; doi:10.1002/dad2.70397)
Supplement: Supplementary file 8 — Supporting Information [file DAD2-18-e70397-s013.docx]

### **Table A2: Summary of missing data***

| **Variable** | **Missing (%)** |
| --- | --- |
| ***Eligibility*** |  |
| Prevalent hearing aid prescription at cohort entry | 7% |
| Self-reported hearing impairment | 9% |
| ***Treatment*** |  |
| New hearing aid prescription (ASPREE year 3) | 13% |
| Hearing aid use (ASPREE year 3) | 13% |
| ***Outcomes*** |  |
| Follow-up biomarkers of ADRD* | 56% |
| ***Covariates*** |  |
| Age | 0% |
| Gender | 0% |
| Race | <1% |
| Education | <1% |
| Income | 15% |
| Socioeconomic status (socioeconomic index for areas) | <1% |
| Baseline biomarkers of ADRD | 24% |
| Difficulty hearing in quiet room | 6% |
| Difficulty hearing in crowded room | 6% |
| 4-Frequency pure tone average | 90% |
| Tinnitus | 15% |
| Systolic blood pressure | 0% |
| Body mass index | <1% |
| eGFR | 3% |
| Liver function | 27% |
| History of cancer | <1% |
| History of diabetes | <1% |
| Antihypertensive use | 0% |
| Polypharmacy | 0% |
| Frailty | 0% |
| Visual function | 3% |
| Chronic kidney disease | 8% |
| Smoking status | 0% |
| Alcohol consumption | 0% |
| APOE-e4 | 14% |
| Typical sleep duration | 2% |
| 3MS Overall score | 0% |
| HVLT-R Delayed recall | <1% |
| CES-D Overall score | <1% |
| SF-12 Mental component score | 0% |
| SF-12 Physical component score | 0% |

* The proportion of missing data in those who survived until the time that follow-up blood draw commenced and therefore were included in the analysis sample.
