# Supplementary material for: The effect of treating hearing loss with hearing aids on plasma biomarkers of Alzheimer's disease and related dementias
Source: Alzheimers Dement (Amst). 2026 Jun 23;18(2):e70397. doi: 10.1002/dad2.70397 (PMC13290640; doi:10.1002/dad2.70397)
Supplement: Supplementary file 9 — Supporting Information [file DAD2-18-e70397-s011.docx]

### **Table A3: Pre-specified auxiliary variables and imputation model specification**

| **Covariate** | **Available ASPREE or ALSOP measures** | **ASPREE follow-up year** | **Functional form** | **Product terms** |
| --- | --- | --- | --- | --- |
| ***Baseline*** |  |  |  |  |
| Baseline confounders | See eTable 1 | Baseline | Quadratic terms for continuous/ordinal variables, categorical otherwise | All two-way exposure-covariate, exposure-outcome, outcome-covariate, and covariate-covariate product terms for treatment, gender, education, baseline 3MS, baseline HVLT-R delayed recall, baseline 4-frequency PTA, difficulty hearing in crowded room, baseline frailty, apoe-e4, and the baseline and follow-up biomarkers of ADRD |
| ***Outcomes*** |  |  |  |  |
| Follow-up biomarkers of ADRD | pTau-181, NfL, GFAP, Aβ42 / Aβ40 | Year 10 | Quadratic | As above |
| ***Longitudinal auxiliary variables*** | |  |  |  |
| Hearing function | Hearing deterioration over last 5 years | Year 3 | Binary | - |
|  | Tinnitus | Year 3 | Quadratic | - |
|  | Difficulty hearing in quiet room | Year 3 | Quadratic | - |
|  | Difficulty hearing in crowded room | Year 3 | Quadratic | - |
|  | Pure tone average of air conduction thresholds at 0.5, 1, 2, and 4 kHz in better ear | Year 3 | Quadratic | - |
| Depression | Center for Epidemiological Studies – Depression total score | Years 3, 6, 9 | Quadratic | - |
| Frailty | Deficit-Accumulation Frailty Index | Years 3, 6, 9 | Quadratic | Treatment, age, gender, education |
| Cardiovascular disease | Myocardial infarction, heart failure, or stroke | Years 3, 6, 9 | Binary | - |
| Cancer | Any cancer diagnosis | Years 3, 6, 9 | Binary | - |
| Physical health | Short-form 12 physical health component score | Years 3, 6, 9 | Quadratic | - |
| Mental health | Short-form 12 mental health component score | Years 3, 6, 9 | Quadratic | - |
| Polypharmacy | Number of medications | Years 3, 6, 9 | Quadratic | - |
| Cognitive function | 3MS overall score | Years 3, 6, 9 | Quadratic | Treatment, age, gender, education |
|  | HVLT-R delayed recall | Years 3, 6, 9 | Quadratic | - |
| Dementia | Dementia diagnosis | Years 3, 6, 9 | Binary | Treatment, age, gender, education |
