# Supplementary material for: The effect of treating hearing loss with hearing aids on plasma biomarkers of Alzheimer's disease and related dementias
Source: Alzheimers Dement (Amst). 2026 Jun 23;18(2):e70397. doi: 10.1002/dad2.70397 (PMC13290640; doi:10.1002/dad2.70397)
Supplement: Supplementary file 10 — Supporting Information [file DAD2-18-e70397-s010.docx]

### **Table A4. Skin cancer physical exam negative treatment control**

| **Biomarker & Strategy** | **Estimated mean** | **Estimated mean difference (95% CI)** |
| --- | --- | --- |
| *pTau-181 (pg/mL)* |  |  |
| No exam | 36.0 | Reference |
| Exam | 36.6 | 0.6 (-0.6, 1.9) |
| *Aβ42/Aβ40 x 1000* |  |  |
| No exam | 61.3 | Reference |
| Exam | 61.3 | 0.0 (-1.2, 1.1) |
| *GFAP (pg/mL)* |  |  |
| No exam | 173.7 | Reference |
| Exam | 173.5 | -0.2 (-4.8, 4.4) |
| *NfL (pg/mL)* |  |  |
| No exam | 31.1 | Reference |
| Exam | 31.2 | 0.1 (-0.9, 1.2) |
