# Supplementary material for: The effect of treating hearing loss with hearing aids on plasma biomarkers of Alzheimer's disease and related dementias
Source: Alzheimers Dement (Amst). 2026 Jun 23;18(2):e70397. doi: 10.1002/dad2.70397 (PMC13290640; doi:10.1002/dad2.70397)
Supplement: Supplementary file 11 — Supporting Information [file DAD2-18-e70397-s012.docx]

### **Table A5. Estimated effects on mean difference scale, using audiometry data to emulate moderate or greater hearing impairment***

| **Biomarker & Strategy** | **Estimated mean** | **Estimated mean difference (95% CI)** |
| --- | --- | --- |
| **First target trial** |  |  |
| *pTau-181 (pg/mL)* |  |  |
| No HA prescription | 37.1 | Reference |
| HA prescription | 39.3 | 2.1 (-0.8, 5.0) |
| *Aβ42/Aβ40 x 1000* |  |  |
| No HA prescription | 61.5 | Reference |
| HA prescription | 61.3 | -0.2 (-3.4, 3.0) |
| *GFAP (pg/mL)* |  |  |
| No HA prescription | 180 | Reference |
| HA prescription | 176 | -4.1 (-16.6, 8.3) |
| *NfL (pg/mL)* |  |  |
| No HA prescription | 34 | Reference |
| HA prescription | 33 | -0.4 (-3.2, 2.5) |
|  |  |  |
| **Second target trial** |  |  |
| *pTau-181 (pg/mL)* |  |  |
| No HA initiation | 37.1 | Reference |
| Initiate using HAs rarely/sometimes | 38.7 | 1.6 (-1.3, 4.5) |
| Initiate using HAs often/always | 40.6 | 3.5 (-0.7, 7.7) |
| *Aβ42/Aβ40 x 1000* |  |  |
| No HA initiation | 61.4 | Reference |
| Initiate using HAs rarely/sometimes | 61.1 | -0.3 (-2.7, 2.1) |
| Initiate using HAs often/always | 61.0 | -0.5 (-5.4, 4.5) |
| *GFAP (pg/mL)* |  |  |
| No HA initiation | 179.4 | Reference |
| Initiate using HAs rarely/sometimes | 176.2 | -3.3 (-14.7, 8.2) |
| Initiate using HAs often/always | 179.0 | -0.4 (-13.5, 12.7) |
| *NfL (pg/mL)* |  |  |
| No HA initiation | 33.5 | Reference |
| Initiate using HAs rarely/sometimes | 34.0 | 0.5 (-2.7, 3.7) |
| Initiate using HAs often/always | 33.3 | -0.1 (-3.2, 2.9) |

***** Better-ear 4-frequency [0.5 – 4 kHz] pure tone average [PTA] of ≥ 30 dBHL. Missing audiometry data was handled by multiple imputation in the full sample.
