# Supplementary material for: The effect of treating hearing loss with hearing aids on plasma biomarkers of Alzheimer's disease and related dementias
Source: Alzheimers Dement (Amst). 2026 Jun 23;18(2):e70397. doi: 10.1002/dad2.70397 (PMC13290640; doi:10.1002/dad2.70397)
Supplement: Supplementary file 12 — Supporting Information [file DAD2-18-e70397-s007.docx]

### **Table A6. Estimated effects on mean difference scale among survivors, after delta adjustment***

|  | **Delta = 0.05 SDs*** | **Delta = 0.25 SDs*** |
| --- | --- | --- |
| **Biomarker & Strategy** | **Estimated mean difference**  **(95% CI)** | **Estimated mean difference**  **(95% CI)** |
| **First target trial** |  |  |
| *pTau-181 (pg/mL)* |  |  |
| No HA prescription | Reference | Reference |
| HA prescription | 1.8 (-0.6, 4.1) | 1.8 (-0.6, 4.1) |
| *Aβ42/Aβ40 x 1000* |  |  |
| No HA prescription | Reference | Reference |
| HA prescription | -0.6 (-2.6, 1.2) | -0.7 (-2.6, 1.2) |
| *GFAP (pg/mL)* |  |  |
| No HA prescription | Reference | Reference |
| HA prescription | -2.2 (-14.5, 10.1) | -2.2 (-14.5, 10.1) |
| *NfL (pg/mL)* |  |  |
| No HA prescription | Reference | Reference |
| HA prescription | 0.1 (-7.8, 8.0) | 0.1 (-7.8, 8.0) |
|  |  |  |
| **Second target trial** |  |  |
| *pTau-181 (pg/mL)* |  |  |
| No HA initiation | Reference | Reference |
| Initiate using HAs rarely/sometimes | 1.7 (-0.9, 4.3) | -1.7 (-0.9, 4.3) |
| Initiate using HAs often/always | 2.3 (-1.0, 5.5) | 2.3 (-1.0, 5.5) |
| *Aβ42/Aβ40 x 1000* |  |  |
| No HA initiation | Reference | Reference |
| Initiate using HAs rarely/sometimes | 0.0 (-2.4, 2.4) | 0.0 (-2.4, 2.4) |
| Initiate using HAs often/always | -1.2 (-3.4, 1.0) | -1.2 (-3.4, 1.0) |
| *GFAP (pg/mL)* |  |  |
| No HA initiation | Reference | Reference |
| Initiate using HAs rarely/sometimes | -2.9 (-7.0, 12.7) | 2.9 (-7.0, 12.7) |
| Initiate using HAs often/always | -1.1 (-12.0, 9.8) | -1.1 (-12.0, 9.8) |
| *NfL (pg/mL)* |  |  |
| No HA initiation | Reference | Reference |
| Initiate using HAs rarely/sometimes | 1.5 (-0.9, 3.9) | 1.5 (-0.9, 3.9) |
| Initiate using HAs often/always | 0.0 (-2.1, 2.2) | 0.0 (-2.1, 2.2) |

*** S**hift constants of 0.05 and 0.25 standard deviations (-0.05 and -0.25 in the case of Aβ42/Aβ40), respectively, were added to each imputed outcome value for participants who were lost to follow-up.
