# Supplementary material for: The effect of treating hearing loss with hearing aids on plasma biomarkers of Alzheimer's disease and related dementias
Source: Alzheimers Dement (Amst). 2026 Jun 23;18(2):e70397. doi: 10.1002/dad2.70397 (PMC13290640; doi:10.1002/dad2.70397)
Supplement: Supplementary file 13 — Supporting Information [file DAD2-18-e70397-s009.docx]

### **Table A7. Estimated effects on mean difference scale among survivors, without adjustment for multiply imputed audiometric hearing loss**

| **Biomarker & Strategy** | **Estimated mean** | **Estimated mean difference (95% CI)** |
| --- | --- | --- |
| **First target trial** |  |  |
| *pTau-181 (pg/mL)* |  |  |
| No HA prescription | 35.7 | Reference |
| HA prescription | 37.7 | 1.9 (-0.5, 4.3) |
| *Aβ42/Aβ40 x 1000* |  |  |
| No HA prescription | 61.4 | Reference |
| HA prescription | 60.7 | -0.7 (-2.4, 1.0) |
| *GFAP (pg/mL)* |  |  |
| No HA prescription | 174.6 | Reference |
| HA prescription | 171.6 | -3.0 (-11.9, 5.8) |
| *NfL (pg/mL)* |  |  |
| No HA prescription | 31.1 | Reference |
| HA prescription | 31.1 | 0.0 (-1.7, 1.8) |
|  |  |  |
| **Second target trial** |  |  |
| *pTau-181 (pg/mL)* |  |  |
| No HA initiation | 35.8 | Reference |
| Initiate using HAs rarely/sometimes | 37.3 | 1.5 (-0.7, 3.8) |
| Initiate using HAs often/always | 38.3 | 2.5 (-0.7, 5.7) |
| *Aβ42/Aβ40 x 1000* |  |  |
| No HA initiation | 61.5 | Reference |
| Initiate using HAs rarely/sometimes | 61.4 | -0.1 (-2.2, 2.0) |
| Initiate using HAs often/always | 60.2 | -1.2 (-3.6, 1.1) |
| *GFAP (pg/mL)* |  |  |
| No HA initiation | 174.3 | Reference |
| Initiate using HAs rarely/sometimes | 174.9 | 0.56 (-7.9, 9.0) |
| Initiate using HAs often/always | 172.2 | -2.2 (-12.3, 7.8) |
| *NfL (pg/mL)* |  |  |
| No HA initiation | 31.1 | Reference |
| Initiate using HAs rarely/sometimes | 32.1 | 0.9 (-1.2, 3.1) |
| Initiate using HAs often/always | 31.0 | -0.1 (-1.9, 1.7) |
