# Supplementary material for: The effect of treating hearing loss with hearing aids on plasma biomarkers of Alzheimer's disease and related dementias
Source: Alzheimers Dement (Amst). 2026 Jun 23;18(2):e70397. doi: 10.1002/dad2.70397 (PMC13290640; doi:10.1002/dad2.70397)
Supplement: Supplementary file 14 — Supporting Information [file DAD2-18-e70397-s015.docx]

### **Table A8. Estimated effects on mean difference scale, estimated using multivariate adaptive regression splines**

| **Biomarker & Strategy** | **Estimated mean** | **Estimated mean difference (95% CI)** |
| --- | --- | --- |
| **First target trial** |  |  |
| *pTau-181 (pg/mL)* |  |  |
| No HA prescription | 35.9 | Reference |
| HA prescription | 37.5 | 1.5 (-0.8, 3.8) |
| *Aβ42/Aβ40 x 1000* |  |  |
| No HA prescription | 61.5 | Reference |
| HA prescription | 60.6 | -0.8 (-3.6, 1.9) |
| *GFAP (pg/mL)* |  |  |
| No HA prescription | 176.0 | Reference |
| HA prescription | 172.8 | -3.2 (-95.2, 88.8) |
| *NfL (pg/mL)* |  |  |
| No HA prescription | 31.4 | Reference |
| HA prescription | 31.4 | 0.0 (-2.4, 2.4) |
|  |  |  |
| **Second target trial** |  |  |
| *pTau-181 (pg/mL)* |  |  |
| No HA initiation | 35.9 | Reference |
| Initiate using HAs rarely/sometimes | 36.8 | 0.9 (-1.3, 3.0) |
| Initiate using HAs often/always | 38.0 | 2.1 (-1.0, 5.2) |
| *Aβ42/Aβ40 x 1000* |  |  |
| No HA initiation | 61.6 | Reference |
| Initiate using HAs rarely/sometimes | 61.0 | -0.6 (-2.4, 1.2) |
| Initiate using HAs often/always | 60.2 | -1.4 (-4.2, 1.5) |
| *GFAP (pg/mL)* |  |  |
| No HA initiation | 174.9 | Reference |
| Initiate using HAs rarely/sometimes | 176.5 | 1.7 (-32.9, 36.2) |
| Initiate using HAs often/always | 172.9 | -1.9 (-14.9, 11.0) |
| *NfL (pg/mL)* |  |  |
| No HA initiation | 31.2 | Reference |
| Initiate using HAs rarely/sometimes | 32.9 | 1.7 (-1.3, 4.7) |
| Initiate using HAs often/always | 31.1 | -0.1 (-2.3, 2.0) |
